# Using the TrueLoo Smart Device to Record Toileting Sessions in Older Adults: Retrospective Validation and Acceptance Study

**DOI:** 10.2196/50856

**Published:** 2024-05-27

**Authors:** Jordan Glenn, Parmoon Sarmadi, Paul Cristman, Gabrielle Kim, Ting-Hsuan Lin, Vikram Kashyap

**Affiliations:** 1 Department of Health, Human Performance and Recreation University of Arkansas Fayetteville, AR United States; 2 Toi Labs, Inc San Francisco, CA United States

**Keywords:** activities of daily living, toileting habits, bowel movements, elder care, smart toileting, monitoring technology

## Abstract

**Background:**

Because of the relationship between independent living and activities of daily living, care teams spend significant time managing assisted living residents’ toileting problems. Recently, the TrueLoo was developed as a connected toilet seat to automatically log and monitor toileting sessions.

**Objective:**

This study aimed to demonstrate the validity of the TrueLoo to (1) record and identify toileting sessions with regard to stool and urine events; (2) compare the results with the person-reported, standard-of-care methods; and (3) establish metrics of user acceptability and ease of use in a assisted living facility population.

**Methods:**

We used two phases: (1) initial development of the TrueLoo algorithms to accurately identify urine and stool events and (2) evaluation of the algorithms against person-reported, standard-of-care methods commonly used in assisted living facilities. Phase 2 analyzed data over a 3-day period from 52 devices. Participants’ age ranged from 63 to 101 (mean 84, SD 9.35) years. Acceptability and ease-of-use data were also collected.

**Results:**

Regarding the development of the TrueLoo algorithm for urine assessment, sensitivity and specificity of 96% and 85% were observed when evaluating a gold-standard labeled data set, respectively (*F*_1_-score=0.95). For stool, sensitivity and specificity of 90% and 79% were observed, respectively (*F*_1_-score=0.85). Regarding the TrueLoo algorithm in assisted living settings, classification performance statistics for urine assessment revealed sensitivity and specificity of 84% and 94%, respectively (*F*_1_-score=0.90), and for stool, 92% and 98%, respectively (*F*_1_-score=0.91). Throughout the study, 46 person-reported instances of urine were documented, compared with 630 recorded by the TrueLoo. For stool events, 116 person-reported events were reported, compared with 153 by the TrueLoo. This indicates that person-reported events were captured 7% (46/630) of the time for urine and 76% (116/153) of the time for stool. Overall, 45% (32/71) of participants said that the new toilet seat was better than their previous one, 84% (60/71) reported that using the TrueLoo was easy, and 99% (69/71) said that they believed the system could help aging adults. Over 98% (69/71) of participants reported that they would find alerts related to their health valuable and would be willing to share this information with their doctor. When asked about sharing information with caregivers, 66% (46/71) reported that they would prefer the TrueLoo to send information and alerts to their caregiver, as opposed to the participant having to personally communicate those details.

**Conclusions:**

The TrueLoo accurately recorded toileting sessions compared with standard-of-care methods, successfully establishing metrics of user acceptability and ease of use in assisted living populations. While additional validation studies are warranted, data presented in this paper support the use of the TrueLoo in assisted living settings as a model of event monitoring during toileting.

## Introduction

The global population is aging rapidly, and by the year 2050, the number of people aged 60 years and older will drastically increase to over 2 billion [1]. Concurrent with this increase in the number of older individuals, life expectancy is projected to increase in the coming decades [2]. However, as is often the case with advanced aging, this increased life expectancy will almost certainly be associated with accompanying morbidities and their associated costs to individuals and health systems [3,4].

These aging demographics will increase the burden of care in assisted living facilities. As part of their regular responsibilities, care staff in these facilities spend significant time and effort documenting and managing their residents’ toileting habits and events [5,6]. This also means they play a key role in the assessment and prevention of toileting-related issues.

While toileting measurement and assistance is an important part of management in assisted living facilities, there is little evidence on how these individuals actually manage bowel problems and the quality of the interventions performed [7]. Furthermore, to track these events and monitor for issues, staff often rely on human reporting. This can come in the form of second-hand information from the resident or the staff member’s own memory when recording multiple residents’ toileting habits at once near the end of a shift; as a result, these methods are incomplete and prone to error.

Because of the relationship between independent living and activities of daily living [8], care teams spend a significant amount of time managing residents’ bowel and bladder problems. Besides common bladder issues such as urinary tract infections [9], bowel problems such as constipation [10], diarrhea [11], and fecal incontinence [12] are highly prevalent among assisted living residents and present several challenges for care staff with regard to monitoring and reporting. Furthermore, these types of events cannot be identified without proper monitoring and recording of toileting events. Recently, the TrueLoo (TL; Toi Labs) was developed as a connected toilet seat to automatically log and monitor toileting sessions, removing the burden from the patient or facility staff. Reporting and monitoring stool and urine characteristics is shown to notably improve the quality of care that residents in assisted living facilities receive, especially those living with multiple comorbidities [13]. The TrueLooprovides concurrent monitoring of toileting sessions, providing caregivers with data to improve their clinical decision-making through evidence-based technology.

Previous investigations and review papers have evaluated the efficacy, practicality, and use of smart toilet seats as a model to longitudinally monitor individual toileting habits. Initial work provided evidence of usability and proof of concept for such a design through the use of a colorimetric assay tracing red-green-blue values from images of urinalysis strips [14]. They also included cameras to collect “analprints” used as unique identifiers. However, there were limitations related to device scalability as well as privacy from upward facing cameras; nevertheless, this was important work to demonstrate functionality of the process [14]. Further work demonstrated the ability to use an image-based data set to classify stool according to the well-established Bristol Stool Form Scale [15], demonstrating the feasibility of using such a technique to automatically evaluate samples [16]. However, while the groundwork for using smart toileting technology has been demonstrated, no data currently exist demonstrating the efficacy and applicability of accurately capturing and evaluating toileting events in a real-world setting.

Therefore, the purpose of this retrospective study was to demonstrate the validity of the TrueLooto (1) record and identify toileting sessions with regard to stool and urine events; (2) compare the results with the person-reported, standard-of-care methods; and (3) establish metrics of user acceptability and ease of use in a assisted living facility population. We hypothesized that the TrueLoowould demonstrate significant improvements in the event capture of toileting sessions compared with the current reporting systems, while being well accepted in the target population.

## Methods

### Ethical Considerations

This was a retrospective analysis, and all procedures were approved by the Western Institutional Review Board (TLSD-001); the study was conducted in accordance with the Declaration of Helsinki.

### Subject Demographics and Recruitment

The participants in the study were aged between 63 and 101 (mean 84, SD 9.35) years. All study participants were from a skilled nursing facility located in Dallas, Texas. Of the 52 participants, 27 (52%) were female. The eligible participants were contacted by community staff, and all individuals who agreed to participate in this investigation were provided with an extensive overview of the product and its capabilities; there were no residents who declined participation. The use of the technology was noted in each resident’s care plan, and community staff were extensively trained by Toi Labs on the capabilities of the product. Finally, Toi Labs provided additional materials to the community to distribute to residents and their families to address concerns about privacy protocols, product overview questions, or other questions regarding the reporting system.

### Inclusion and Exclusion Criteria

This study was conducted as a retrospective analysis, and, as such, there was no participant consent required. However, to participate in the original commercial program, each participant was required to meet all of the following inclusion criteria: (1) willing to participate and provide consent for the program, (2) aged 55 years or older, (3) a resident of the assisted living facility where the data were collected, and (4) had regular access to a TrueLoo.

Additionally, participants satisfying any of the following exclusion criteria were precluded from participation in the study: (1) unwilling or unable to accept the requirements associated with installing the TrueLooin their residence, including power and Wi-Fi connectivity, and (2) used certain types of toileting assistance devices that, at this time, are not compatible with the TrueLoo(eg, padded toilet seat risers).

### Study Design

This study was conducted in two phases:

1. Development of the TrueLoo algorithms to accurately identify urine and stool events
2. Evaluation of the algorithms against the real-world person-reported, standard-of-care methods commonly used in assisted living facilities

It is suggested that traditional methods for developing robust evidence are incongruent with the agile approach commonly used in software development, as there is often an incongruence between the length of traditional registered controlled trials and the development and update cycle for software [17]. Given the agreement that more traditional approaches present fundamental limitations for proper evidence generation for digital health solutions, this 2-phase approach allowed for the controlled development of the algorithms from real-world data collected as part of a commercial program and then analyzed and evaluated retrospectively for efficacy.

For phase 1, details surrounding the development of the initial algorithm for identifying stool and urine events are described below. For phase 2 (real-world data collection), participants were enrolled independent of symptoms, disease diagnosis, or state. Furthermore, in an effort to attain real-world applicability, there was no stratification of the population by sex, age, race, or disease severity. Each participant was monitored in their residences within their respective community. For each resident, a TrueLootoilet seat was installed in their private bathroom along with an initial health assessment to collect demographic information and any preexisting conditions. During this phase, 52 devices were deployed and reviewed for a 3-day period. Data were collected on each of the 52 participants using the TrueLoo.

### Measures

#### Development of the TrueLooAlgorithm for Identifying Urine and Stool Events

To verify urine and stool events that were accurately captured in the development of the algorithm, a panel of board-certified, gastroenterologist, subject-matter experts were first enlisted to create a “gold-standard” database. The subject-matter experts created a rule set for image annotators, who were trained to accurately identify image content. These image annotators provided our ground truth and produced a labeled data set that was then used to train the machine learning algorithms before being run on the full data set. The applied labels were used to create digital biomarkers for toileting event imagery. As of this investigation, more than 10,000 sessions (times people have used the toilet) were labeled, with more than 40,000 images. A session was activated when a user is in proximity to or sitting on the toilet seat. Each session comprises multiple images that depict the physical characteristics of urine and stool seen throughout the session in a time series.

The TrueLoo algorithms were developed using an existing large image model that is pretrained to extract fundamental image features such as shapes, colors, and textures. This model is then trained on proprietary image data collected by the TrueLoo that have been human annotated with label taxonomy of over 20 contextual labels that include relevant health metrics such as the Bristol Stool Scale and nonhealth metric such as toilet cleaning. The labeled data set is used to refine the large image model to create the proprietary TrueLoo algorithms.

**Figure 1:**
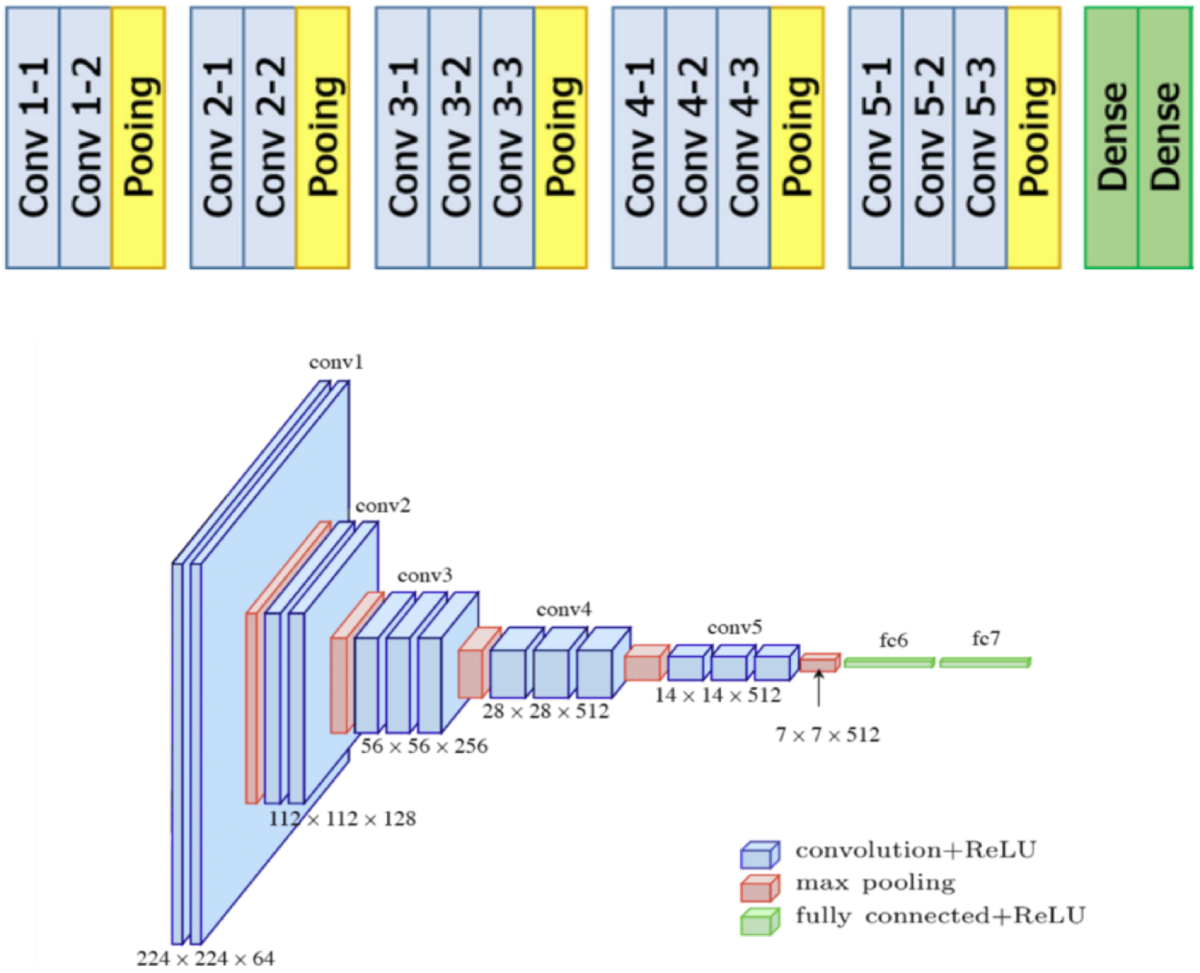
Convolutional neural network architecture. Conv: convolutional block; ReLu: rectified linear unit.

A classic deep learning network structure was used for the neural network architecture, as illustrated in Figure 1. The network consists of 5 convolutional blocks with max pooling for feature learning and extraction, followed by 2 dense layers. A sigmoid activation function on the final layer is used for the final multilabel classification task and a binary cross-entropy loss function. This architecture has been very successful in traditional image classification tasks, and pretrained weight configurations based on classic benchmarks are readily available, making it an ideal candidate for fine-tuning and transfer learning.

#### Detailed Working Description of the TrueLoo Technology

**Figure 2:**
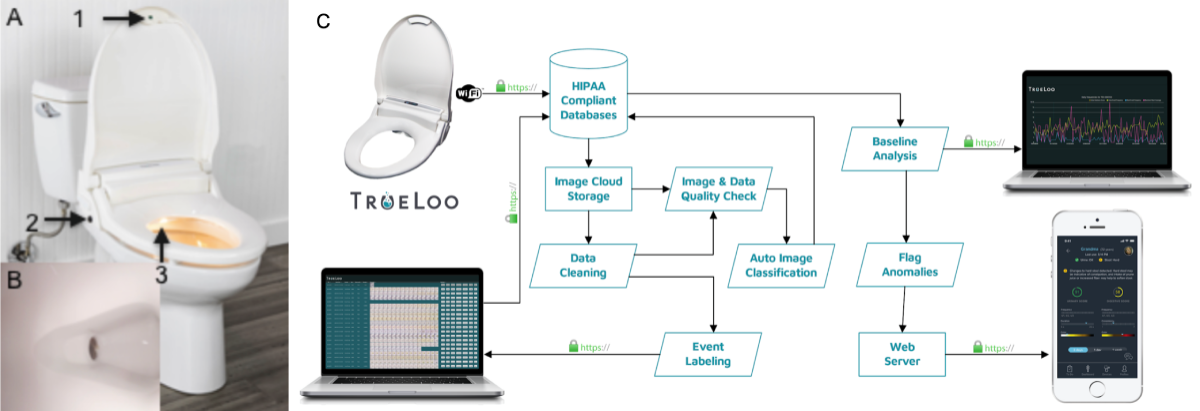
The TrueLoo platform and associated dataflow. (A) Image of TrueLoo installed on a toilet: (1) user presence sensor for risers and male standing urinating; (2) multifunction guest button: excludes guests who may use the toilet; (3) bowl illumination, optical system that scans the contents of the toilet bowl. (B) Example frame captured by TrueLoo. (C) TrueLoo software block diagram; data captured by TrueLoo is uploaded to HIPAA-compliant databases and data flow begins. HIPAA: Health Insurance Portability and Accountability Act.

The TrueLoo consists of 2 parts: a hardware component that is delivered as a replacement toilet seat and a software system for analysis and reporting. Figure 2A shows a photograph of the TrueLoo seat with features called out and an image (example in Figure 2B) captured from the optical system. The TrueLoo seat has 2 user presence sensors: a contact sensor bound to the seat with no visible sign to the user and a noncontact time-of-flight distance sensor (Figure 2A.1) that activates when the user does not sit on the seat (ie, standing while urinating). By using the 2 sensors, the system distinguishes between standing and seated events as well as nonevent classification. The rear housing is used to mount the optical system and support electronics. The bowl is illuminated (Figure 2A.3) uniformly by red-green-blue-white LEDs to control color balance and some narrow band imaging illuminating with only 1 color. This allows for consistent imaging conditions for all currently encountered toilet geometries. Not shown in the image is the red-green-blue 8-megapixel manual focused camera and needed control and communication electronics. The system is powered by a single-board computer with integrated Wi-Fi communications for transmitting the images. The TrueLoo seat has a guest button (Figure 2A.2) to disable the system if a guest needs to use the toilet. The guest button automatically resets after each use. No images are recorded from guest events; however, they are registered in the database as an activation of the TrueLoo. Not shown in the figure is the cable routing using a conduit to fix the cable to the wall and connect the unit to power using a wall mount type AC-DC transformer at the outlet allowing for a long cable run with a low-voltage thin wire; this setup does not require a new outlet or the replacement or recharging of batteries. The seat is fixed to the toilet using a standard commercial mounting system for replacement toilet seats. After the TrueLoo seat is installed, it requires minimal to no ongoing maintenance, other than ensuring that the optics stay clean.

When a user activates the TrueLoo by sitting on the seat or standing in front of the toilet, the system activates an event and immediately starts imaging at 1.2 frames per second. The TrueLoo continuously captures images of the bowl for the duration of time the user is seated or standing in front of the device. Immediately after the event is finished the images are transferred via Wi-Fi to Health Insurance Portability and Accountability Act (HIPAA)–compliant servers for storage and analysis (Figure 2C). Duplicate images are not uploaded but are registered to ensure correct time sequencing and more.

Because of the non–battery power and Wi-Fi connectivity configuration, data could be logged and monitored to ensure that the device was connected and working properly. The device can save approximately 1 week of data locally and resume uploading data if and when a Wi-Fi connection is compromised. This capability provides added capture and integrity in the event of a prolonged Wi-Fi outage.

#### Tracking of Toileting Events in Assisted Living Facilities

As previously mentioned, person-reported methods of bowel movement and urinary event tracking are considered the standard of care in assisted living settings. For phase 2 of this study, the same annotators retrospectively labeled toileting images captured through a commercial engagement, creating a real-world ground truth data set. We then analyzed recorded TL-captured events through the algorithm developed in phase 1, comparing them with the person-reported events captured by facility staff, broken down into urinations and bowel movements.

The current practice for reporting toileting events among facility staff involves documentation at the end of a shift, sometimes manually (pen to paper in a chart), or other times in the electronic medical record. The analysis done was for a community that reports this information in the electronic medical record. The data recorded in bowel and bladder logs are often inadequate, with limited description. For example, the logging for urination only allows facility staff to notate “Void? Yes or No.” Many of the entries are listed as “Not Applicable.”

Bowel elimination questions include (1) the size of bowel movement (small, medium, large, resident not available, resident refused, and not applicable), (2) consistency of bowel movement (formed or normal, loose or diarrhea, constipation or hard, putty like, resident not available, resident refused, and not applicable), and (3) bowel continence (continent, incontinent, no bowel movement, continence not rated due to ostomy, resident not available, resident refused, and not applicable).

The TrueLoo system automatically classifies on an established scale based on the Bristol Stool Scale [15]. The following classifications were used to inform the TrueLoo algorithms: separate, hard lump nut-like stool; soft blobs with distinct edges; sausage-like stool with surface cracks; lumpy, sausage-like stool; smooth, sausage-snake stool; fluffy, mushy stool with ragged edges; and watery liquid, no solid stool.

Most of the data captured by the TrueLoo are supplemental when benchmarked against the information recorded by facility staff through the logs. As seen in our analysis, although the elimination questions in the electronic records have the potential to capture the characteristics of stool and urine, they are not accurately completed by care staff, if at all.

### Data Safety and Integrity

Protocols are included as a part of the TrueLoo implementation’s standard process to ensure that privacy is maintained at all times through the data collection process.

1. The TrueLoo imaging system faces down into the toilet bowl and is designed specifically to scan stool and urine; it does not capture any body parts.
2. User information is completely deidentified, from data capture to analysis.
3. The seat itself does not carry the name or location of the user.
4. All deidentified data are stored internally—none of the data that are viewed or used are associated with any sort of identifier—and are completely anonymized.

Secure servers and connections allow reports to only be shared with onsite care teams tasked with caring for the user. The reports can be shared with outside care partners (such as physicians) or families. Toi Labs’ team members review and validate reports to ensure compliance of privacy protocols and offer the best service and care to users.

Information is not identifiable on capture. Multiple protocols are in place throughout data capture to ensure that the data remain completely deidentified. No TrueLoo seat or its data are directly linked to a resident’s name or room number. This information is secured internally and is only used when providing reports to care teams. Otherwise, all data that are captured and uploaded are deidentified and anonymized. Secure servers and connections allow reports to only be shared with onsite care teams tasked with caring for the user. Upon the authorization of the user or their responsible party, the reports can be shared with outside care partners or families as they see fit. The reports are not distributed to anyone outside of the circle of care of the user.

### Participant Satisfaction and Acceptability

At the end of the study, a survey about user satisfaction was provided to all participants to evaluate acceptability and comfort with the use of the TrueLoo. This survey can be found in Multimedia Appendix 1.

### Data Analyses

Python version 3.9 (Python Software Foundation) and R version 4.1.3 (R Foundation) were used to conduct all analyses. Descriptive statistics were calculated for age and sex. For the development of the initial algorithms, sensitivity, specificity, precision, recall, *F*_1_-scores, and receiver operating characteristic (ROC) area-under-the-curve (AUC) were calculated to evaluate the classification performance of stool and urine events. Bootstrapping was used to calculate 95% confidence intervals for AUC.

For evaluation of the algorithm in real-world evidence (RWE) settings, sensitivity, specificity, precision, recall, *F*_1_-scores, and ROC analyses were performed. Overall results were compared with the person-reported events as a percent to determine effectiveness to the standard of care.

## Results

### Data Characteristics

Throughout the period involved in the study’s retrospective analysis, a total of 645 toileting sessions were recorded by the TrueLoo. Of the 645 total recorded events, 630 included urine and 153 included stool. There was overlap between the number of sessions containing both urine and stool. In this investigation, the average age of the participants was 84 (SD 9) years, with 52% (27/52) of the participants identifying as female. All of the individuals in the setting in question used the TrueLoo for the 3 days as planned. No participants declined the use of the TrueLoo.

### Development of the TrueLoo Algorithm for Identifying Urine and Stool Events

**Figure 3:**
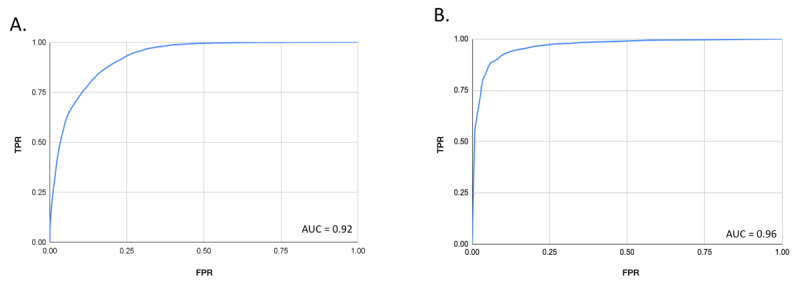
Receiver operating characteristic area-under-the-curve (AUC) analysis for the TrueLoo algorithm to identify (A) urine and (B) stool events. fpr: false positive rate; tpr: true positive rate.

With regard to the development of the TrueLoo algorithm, classification performance statistics for all urine and stool events can be found in Table 1, and ROC curves can be found in Figure 3. For urine assessment, AUC was 0.92, with sensitivity and specificity of 96% and 85% observed, respectively. For stool, AUC was 0.96, with sensitivity and specificity of 90% and 79% observed, respectively.

**Table 1:** Classification performance of the TrueLoo algorithm for detecting urine and stool events.

Event	Precision	Recall	Sensitivity	Specificity	*F*_1_-score
Urine	0.94	0.96	0.96	0.85	0.95
Stool	0.80	0.90	0.90	0.79	0.85

### Toileting Sessions Compared With Standard of Care

**Figure 4:**
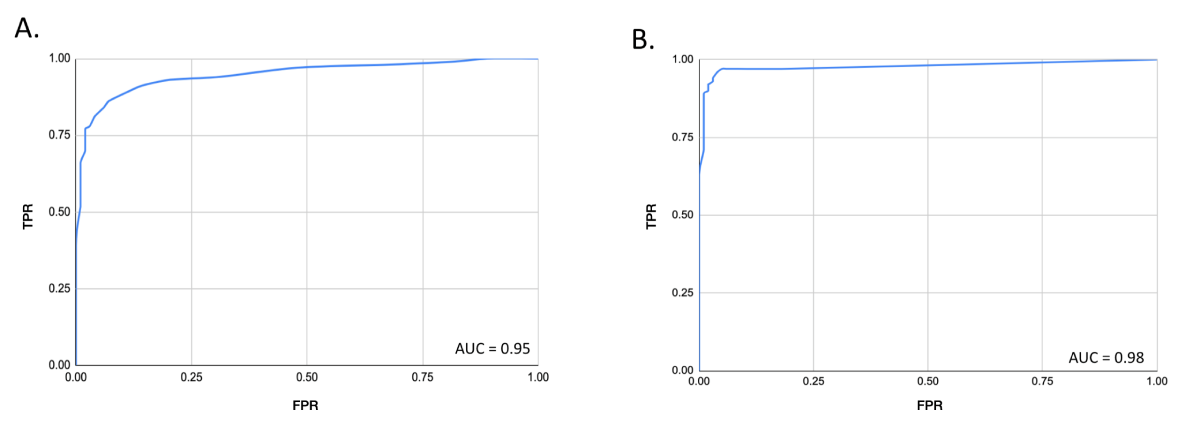
Receiver operating characteristic area-under-the-curve (AUC) analysis for the TrueLoo algorithm in real-world evidence settings (ie, standard of care) to identify (A) urine and (B) stool events. fpr: false positive rate; tpr: true positive rate.

With regard to the evaluation of the TrueLoo algorithm in RWE settings, classification performance statistics for all events can be found in Table 2, while ROC curves can be found in Figure 4. For urine assessment, AUC was 0.95, with sensitivity and specificity of 84% and 94% observed, respectively. For stool, AUC was 0.98, with sensitivity and specificity of 92% and 98% observed, respectively.

**Table 2:** Classification performance of TrueLoo for detecting urine and stool events in assisted living settings (real-world evidence analysis).

Event	Precision	Recall	Sensitivity	Specificity	*F*_1_-score
Urine	0.97	0.84	0.84	0.94	0.90
Stool	0.91	0.92	0.92	0.98	0.91

To compare the TrueLoo with the person-reported standard of care, we compared the number of reported instances in the skilled nursing facility with the number of events recorded by the TrueLoo. Over the course of the study, there were 46 person-reported instances of urine documented, compared with 630 by the TrueLoo. For stool events, there were 116 person-reported events compared with 153 events reported by the TrueLoo. This indicates that, when compared with the TL, person-reported events were captured 7% (46/630) of the time for urine and 76% (116/153) of the time for stool.

### Survey Feedback on Acceptability and Ease of Use

A high degree of user satisfaction was found in the exit survey. Overall, 45% (32/71) of participants said the new toilet seat was better than their previous seat, while 46% (33/71) said it was the same. Importantly, 84% (60/71) of participants reported that using the TrueLoo was easy and required no effort; 99% (69/71) said they believed that the monitoring system had the potential to help aging adults.

With regard to the transmitting and interpretation of data, 99% (69/71) of participants reported that they would find alerts related to their health valuable and would also be willing to share this information with their doctor. When asked about sharing information with caregivers, 66% (46/71) reported that they would prefer the TrueLoo to send information and alerts to their caregiver, as opposed to the participant having to personally communicate those details.

## Discussion

### Principal Findings

The purpose of this retrospective study was to demonstrate the validity of the TrueLoo to (1) record and identify toileting sessions with regard to stool and urine events; (2) compare the results with the person-reported, standard-of-care methods; and (3) establish metrics of user acceptability and ease of use in a assisted living facility population. Our hypothesis that the TrueLoo would accurately identify urine and stool events, as well as capture more toileting sessions than were captured via the standard of care, was confirmed. We also confirmed our hypothesis that this method would be well accepted by residents.

### Ability to Record and Identify Toileting Sessions

In this investigation, the TrueLoo algorithm demonstrated high sensitivity, specificity, and accuracy for detecting urine and stool events in real-world settings. Toileting issues such as urinary tract infections [9], constipation [10], diarrhea [11], and fecal incontinence [12] are highly prevalent among assisted living residents; however, if regular monitoring of toileting events are not accurate, it becomes difficult to identify and manage these conditions. Being able to manage these clinical events begins with the identification and monitoring of toileting; if it cannot be tracked, it cannot be measured and therefore cannot be improved.

Previous research investigating the use of smart toileting technology provides an initial look into the feasibility and proof of concept related to successfully executing this type of monitoring model [14-16]. However, these designs and models were limited as it pertains to real-world applicability. Early work used a colorimetric assay tracing red-green-blue values from images of urinalysis strips as well as upward facing cameras to collect “analprints” used as unique identifiers [14]. Based on the recommendation by Ge et al [18], “To enhance data quality, devices should be designed in ways that are physically or psychologically unobtrusive so as not to influence normal toileting behavior,” this initial model becomes problematic as the process can be intrusive and pose risks to data privacy.

### Comparison of TrueLoo With Traditional Standard of Care

The standard of care (facility staff manual reporting) requires an individual to remember each event, manually track the event, and recall specific details about the event that would be relevant to a clinical issue. While this is theoretically feasible, it is unrealistic to expect these individuals to remember specific details and document each event without error throughout the course of a shift. Furthermore, expecting patients to remember and accurately report their own events is difficult due to recall bias and the natural discomfort of discussing one’s own toileting habits [19], especially if the events are different or unusual. This can be combined with the fact that, perhaps unsurprisingly, residents in seniors living facilities report that noninvasive methods of care are preferable to more invasive methods when it comes to the development of toileting programs [19,20]. This concept that there is a reluctance to openly discuss excreta [21,22] has hampered the development and acceptance of smart toilets; however, the use of smart toileting technology, such as the TL, creates a way to seamlessly integrate toileting analyses as part of routine monitoring, serving as a gateway to the digitalization of health care in the home [18].

Through this investigation, we found that facility staff underreport toileting events when compared with the TrueLoo. Given the active versus passive nature of the 2 methods, this is unremarkable, provided the limitations naturally inherent on the time and resources of human monitoring. Not only are facility staff dependent on their own timing for successfully monitoring a resident’s toileting habits, but they are also highly reliant on residents being honest about their own habits in the instances when they cannot be monitored or observed directly [13]. Furthermore, it appears that staff tend to log these data points in batches, often from memory. For example, most sites use a shift system where their staff are working from 6:00 to 14:00 hours, from 14:00 to 23:00 hours, and from 23:00 to 6:00 hours. Based on a single day of data provided by the site from their self-reporting logs (Multimedia Appendix 2), the majority of toileting events are reported toward the end of their respective shift. This is especially evident in the later evening shifts where the majority of reports from the 14:00-23:00 shift were reported from memory within a 2-hour time span. The level of cognitive load required with this method, in addition to their other responsibilities [7], is highly prone to error.

Many assisted living residents require more complex and advanced care compared with the general population, and evidence supports the fact that nursing home clinical outcomes are heavily reliant on geriatric approaches and care leadership. Proper care of these residents requires a multidimensional and specialized approach from facility staff [23,24]; however, recognition for this type of skill and the effort involved is frequently undervalued, thus leading to staff turnover and vacancies [25]. Furthermore, the high prevalence of toileting issues among nursing home residents indicates that there is demonstrable potential for improvement within this population. Such issues among residents are largely related to remediable factors, which can potentially be prevented or improved [7], and current nursing home practices do not adequately address these challenges. Providing a passive monitoring toileting solution that captures these types of data automatically, analyzes them, and transmits them back to the facility can reduce time and discomfort required for staff. This would allow an already underappreciated group [25] to focus on alternative needs in their respective facilities, removing burden and potentially increasing staff morale and attitude. The latter is, perhaps, most important because previous research from assisted living settings has shown that attitudes of care staff toward their organization, residents, and families have a significant effect on the quality of care provided to the residents [26].

### Clinical Relevance in Real-World Settings

As discussed above, current standard-of-care methods are error prone and inconsistent. This inconsistency of monitoring potentially creates a larger issue for identifying critical conditions associated with greater costs and health care needs. Residents in assisted living facilities are at significant risk of developing issues such as urinary tract infections [9] and bowel problems such as constipation [10], diarrhea [11], and fecal incontinence [12], to name a few. These issues cannot be consistently identified without proper monitoring of toileting events. For example, when evaluating the prevalence of inpatient falls in a Michigan community hospital, 45.2% were related to toileting-based issues [27]. Importantly, 82.3% of patients who fell had completed a fall risk assessment before the incidents, indicating minimal relationship between fall assessment and actual falls. These results were further confirmed in a secondary data analysis conducted on 281,865 high-risk falls assessments collected in a multisite study where toileting issues were the third most powerful predictor of falls after “falls in the last 6 months” and “confusion” [28]. Furthermore, a retrospective analysis of falls related to nighttime toileting over a 1-year period found that 34% of falls were related to toileting-related issues [29]. Finally, a cross-sectional report evaluating the association between toileting and falls in older adults admitted to an emergency department discovered that the rate of recurrent falls was significantly higher in a toileting-related falls group than a non–toileting-related falls group [30]. As such, it is critical that toileting issues be addressed upstream to prevent larger, related issues from occurring. The use of the TrueLoo to accurately, consistently, and passively track toileting habits and related issues may provide the necessary feedback to ensure that residents receive the care and attention required to prevent falls from occurring, instead of requiring active response after the fall occurs.

### Acceptability and Ease of Use

There are an ever-growing number of technological solutions offering potential benefits for older adults. However, despite the potential benefits, older adults regularly demonstrate lower adoption rates compared with their younger peers [31-33]. In the older adult population, perceived value, confidence in the ability to learn the technology, and the perceived impact on quality of life are reported to be some of the most robust predictors of willingness to adopt technology [34]. In this investigation, 84% (60/71) participants reported that using the TrueLoo was easy and required no effort (ability to learn) and 99% (69/71) said they believed that the monitoring system had the potential to help aging adults (perceived impact). Additionally, 99% (69/71) of participants reported that they would find alerts related to their health valuable and would also be willing to share this information with their doctor, and 66% (46/71) reported that they would prefer the TrueLoo to send information and alerts to their caregiver, as opposed to the participant having to personally communicate those details (quality-of-life improvements). These data points triangulate to the TrueLoo being not only efficacious but also successfully adopted as a passive monitoring intervention in this age group.

### Strengths and Limitations

The strengths of this study include the process of initially developing the algorithm for identifying stool and urine events against a gold-standard labeled data set and then retrospectively analyzing it in an RWE setting. This methodology allowed for the ability to generate real-world insights into how the TrueLoo could most effectively be used in assisted living facilities. Additionally, the ability to get direct feedback from the end users provides subjective validation, in addition to the efficacy of the TrueLoo to evaluate sessions. There is often a disconnect between clinical efficacy and practical use, which prevents new technology from being properly implemented. While RWE designs are powerful for real-world practicality, there are associated limitations. In this study, there was a lack of ability to control certain parameters (monitoring and reporting habits of facility staff) and collect certain data points (detailed health reports) on the participants. Ameliorating these limitations would change facility workflows, therefore affecting the validity of real-world efficacy. As such, we accept these limitations but recommend that this study be followed up with additional controlled investigations into the clinical efficacy of the TrueLoo. Such controlled investigations would also allow for the evaluation of other indications such as loose or bloody stool or cloudy urine. Given the clinical applicability of these indications, they are a recommended next step for future research.

### Conclusions

In this retrospective validation and acceptance study, we demonstrated the validity of the TrueLoo to record toileting sessions compared with the standard-of-care methods, while categorizing them into clinically relevant events. Additionally, the TrueLoo successfully established metrics of user acceptability and ease of use in assisted living populations. While additional validation studies are warranted, the data presented in this paper support the use of the TrueLoo in assisted living settings as a model of session monitoring during toileting.

## Appendix

Multimedia Appendix 1 Exit survey on user acceptance and satisfaction.

Multimedia Appendix 2 Example chart of a facility’s toileting logging timing throughout a single day.

## Abbreviations

AUC: area under the curve
HIPAA: Health Insurance Portability and Accountability Act
ROC: receiver operating characteristic
RWE: real-world evidence
TL: TrueLoo
