# Supplementary material for: Using the TrueLoo Smart Device to Record Toileting Sessions in Older Adults: Retrospective Validation and Acceptance Study
Source: JMIR Aging. 2024 May 27;7:e50856. doi: 10.2196/50856 (PMC11165284; doi:10.2196/50856)
Supplement: Multimedia Appendix 1 [file aging_v7i1e50856_app1.docx]

# Multimedia Appendix 1: Exit survey on User Acceptance and Satisfaction

1. Name of Participant
2. Name of Community
3. How long did the resident use the new toilet seat?
4. Regarding just your experience with the new toilet seat, how much effort was required to use it?
5. How did the new toilet seat compare to your previous toilet seat?
6. What do you think of the potential of this toilet monitoring system to help older adults?
7. If the system provided you alerts for potential health issues would you find that valuable?
8. If you weren’t feeling well and needed to visit the doctor, would you share information from the toilet seat to help in the diagnosis?
9. Would you feel more comfortable if we sent urgent alerts about your stool or urine to your caregivers instead of you having to tell them?
10. What did you like most about the new toilet seat?
11. What do you think can be improved in the toilet seat?
12. Name of Primary Physician
13. Name of Medical Group
