# Supplementary material for: Using the TrueLoo Smart Device to Record Toileting Sessions in Older Adults: Retrospective Validation and Acceptance Study
Source: JMIR Aging. 2024 May 27;7:e50856. doi: 10.2196/50856 (PMC11165284; doi:10.2196/50856)
Supplement: Multimedia Appendix 2 [file aging_v7i1e50856_app2.docx]

# Multimedia Appendix 2. Example chart of a facility’s toileting logging timing throughout a single day


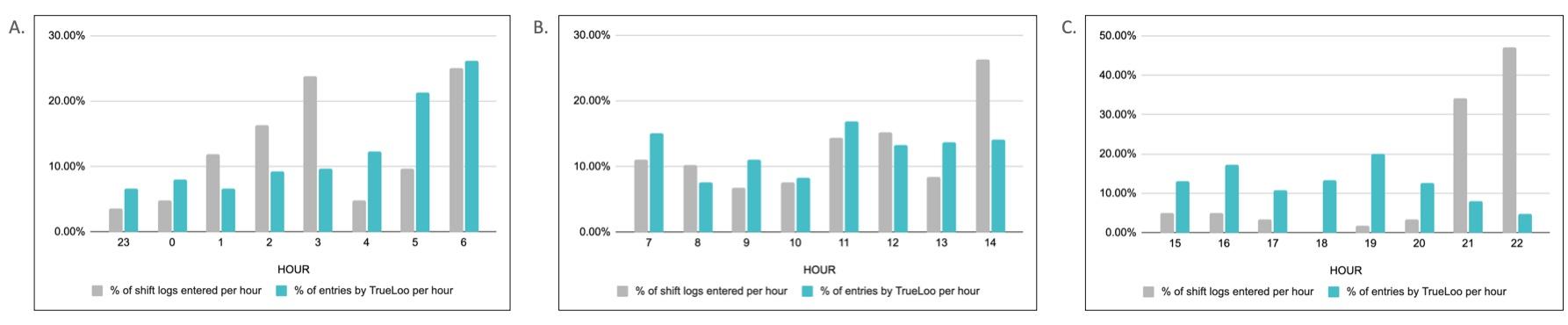


Example chart of a facility’s toileting logging timing throughout a single day. The site utilized a 3-shift system where their staff worked from 6:00-14:00 (a), 14:00-23:00 (b), and 23:00-6:00 (c).
